# Granulomatous mastitis forming a well-defined large mass diagnosed by surgical excision: a case report

**DOI:** 10.1186/s40792-024-02059-6

**Published:** 2024-11-08

**Authors:** Chisaki Hao, Yoshiya Horimoto, Toshitaka Uomori, Akihiko Shiraishi, Gotaro Orihata, Hiroko Onagi, Takuo Hayashi, Junichiro Watanabe, Goro Kutomi

**Affiliations:** 1 Department of Breast Oncology, Faculty of Medicine, Juntendo University, 2-1-1 Hongo, Bunkyo-Ku, Tokyo, 113-0033 Japan; 2 Department of Breast Surgery and Oncology, Tokyo Medical University, 6-7-1 Nishishinjuku, Shinjuku-Ku, Tokyo, 160-0023 Japan; 3 Department of Human Pathology, Faculty of Medicine, Juntendo University, 2-1-1 Hongo, Bunkyo-Ku, Tokyo, 113-0033 Japan; 4 Department of Radiology, Faculty of Medicine, Juntendo University, 2-1-1 Hongo, Bunkyo-Ku, Tokyo, 113-0033 Japan

**Keywords:** Granulomatous mastitis, Idiopathic, Surgical intervention

## Abstract

**Background:**

Granulomatous mastitis is a relatively rare benign inflammatory disease of the breast, but it is sometimes difficult to distinguish from breast cancer by imaging. We experienced a case that was definitively diagnosed as granulomatous mastitis from the surgical specimen. The mass appeared as a large cystic lesion on imaging, which is unusual for granulomatous mastitis, and was initially suspected to be an encapsulated papillary carcinoma.

**Case presentation:**

A 43-year-old woman presented with a painful mass in her right breast. Ultrasonography revealed a cystic mass lesion with internal solid components, with partially indistinct cyst walls and abundant blood flow. Additionally, lymphadenopathy of one axillary lymph node was observed. Magnetic resonance imaging findings showed irregularly spreading enhanced nodules within the cystic lesion, raising the suspicion of encapsulated papillary carcinoma. Although the histological findings from a needle biopsy were consistent with granulomatous mastitis, the possibility of malignancy could not be ruled out based on imaging, prompting a diagnostic probe lumpectomy. However, the surgical specimens did not reveal any tumorous lesions, and we reached a final diagnosis of granulomatous mastitis. Postoperatively, the patient was followed-up without steroid therapy and has been free from recurrence of mastitis for 22 months after surgery.

**Conclusions:**

We report a case of granulomatous mastitis that was detected as a large cystic lesion with a well-defined border on imaging and a definitive diagnosis was made from a surgical specimen.

## Background

Granulomatous mastitis (GM), first described in 1972 by Kessler and Wolloch [1], is a relatively rare benign inflammatory breast disease, with a mean age of 36 years (range, 19–49 years) [2], and most cases have a history of pregnancy or breastfeeding [3]. While trauma, metabolic or hormonal processes, autoimmune factors, and infections are thought to be implicated, the exact etiopathogenesis is unknown [4]. The clinical presentation is often a painful mass lesion with skin erythema, and may be associated with axillary lymphadenopathy [2]. GM is challenging to differentiate from breast cancer based on imaging alone due to overlapping features [5]. On ultrasound, GM often presents as a contiguous, heterogeneously hypoechoic mass (es) with ill-defined margins and tubular extensions [6, 7]. Hypervascularity, detectable by Doppler imaging, is frequently observed [7]. However, these findings are not specific to GM, and are commonly seen in malignancies as well. In GM without specific mass lesions, magnetic resonance imaging (MRI) lacks sufficient specificity to differentiate inflammatory changes (skin thickening/increased vascularity) from malignant tumors, such as inflammatory breast cancer [5, 8, 9]. Therefore, imaging plays a limited role in the definitive diagnosis of GM [10, 11].

In clinical practice, a needle biopsy is often performed primarily to rule out cancer. Although the histopathology of GM has relatively characteristic findings, such as the formation of epithelial granulomas, the diagnosis of this disease is made based on a comprehensive assessment of clinical symptoms, imaging findings and histological findings. In this report, we describe a case of mass-forming GM that was suspected to be malignant on imaging and a definitive diagnosis was only made after surgical resection.

## Case presentation

**Fig. 1:**
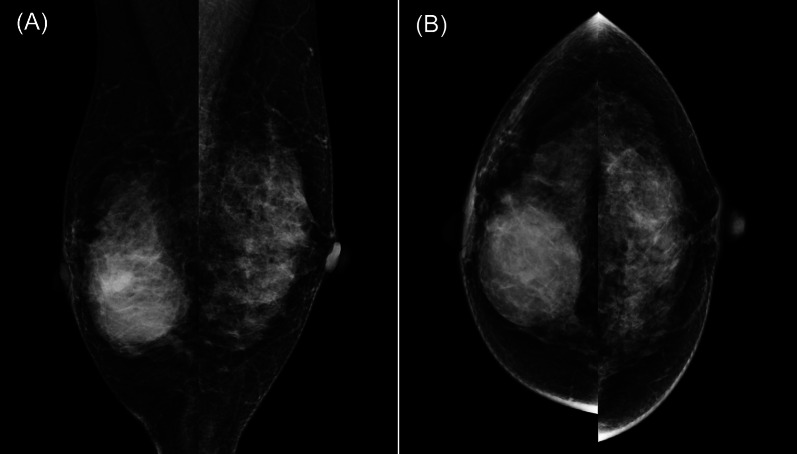
Findings on mammogram**.** **A** Medio-lateral oblique and **B** craniocaudal views. **A** 45-mm isodense, partially indistinct, subintra-areolar mass was observed below the right nipple

**Fig. 2:**
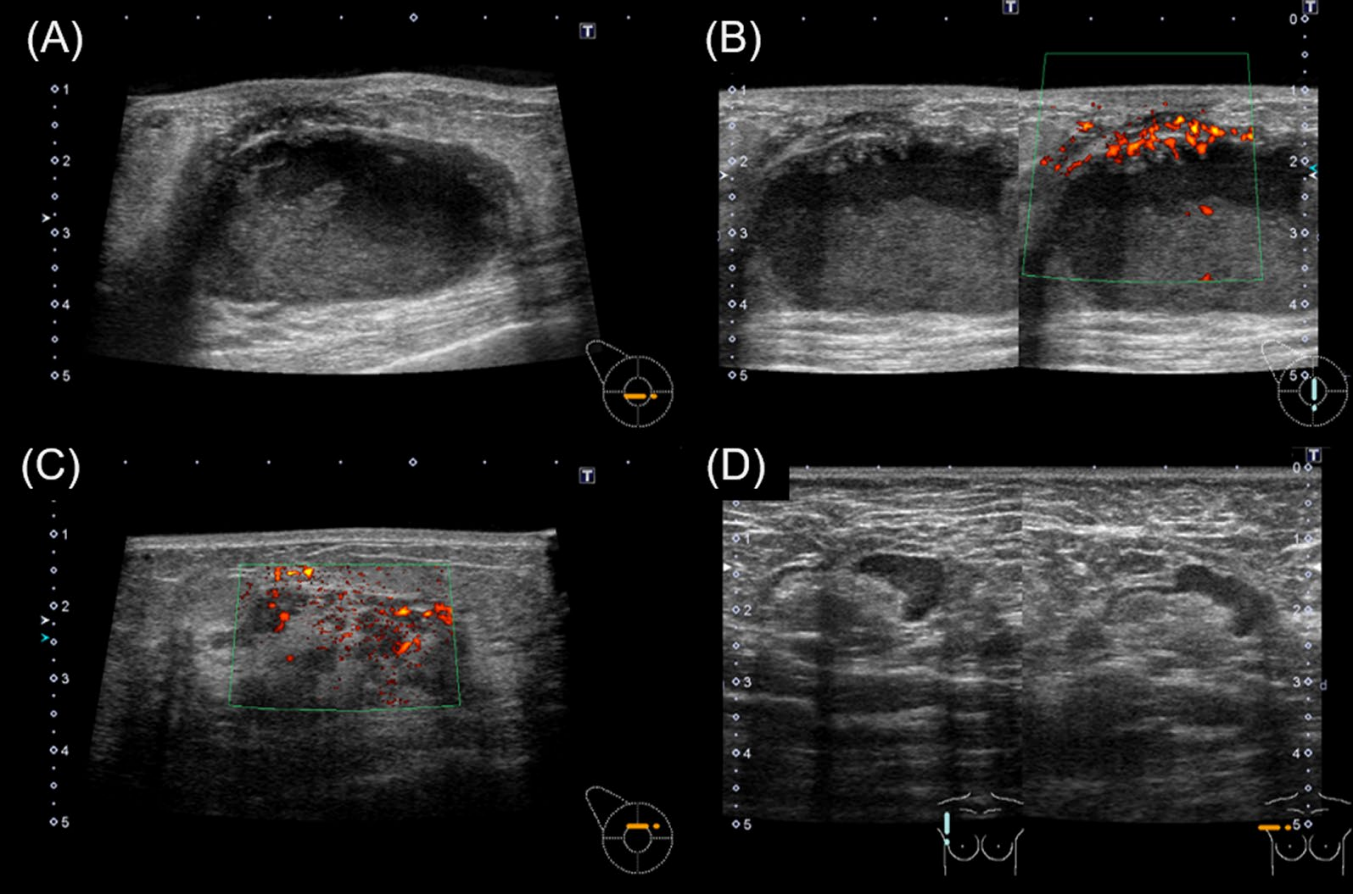
Findings on ultrasound**.** **A** A 48 × 45x24-mm cystic mass with an internal broad-based component was seen just below the right nipple to lower breast. **B** The cyst wall was partially obscured and color Doppler showed abundant blood flow. **C** A hypoechoic area with abundant blood flow was observed just above the main lesion. **D** An enlarged (25 × 23x10 mm) level I axillary lymph node with thick cortical swelling was observed

**Fig. 3:**
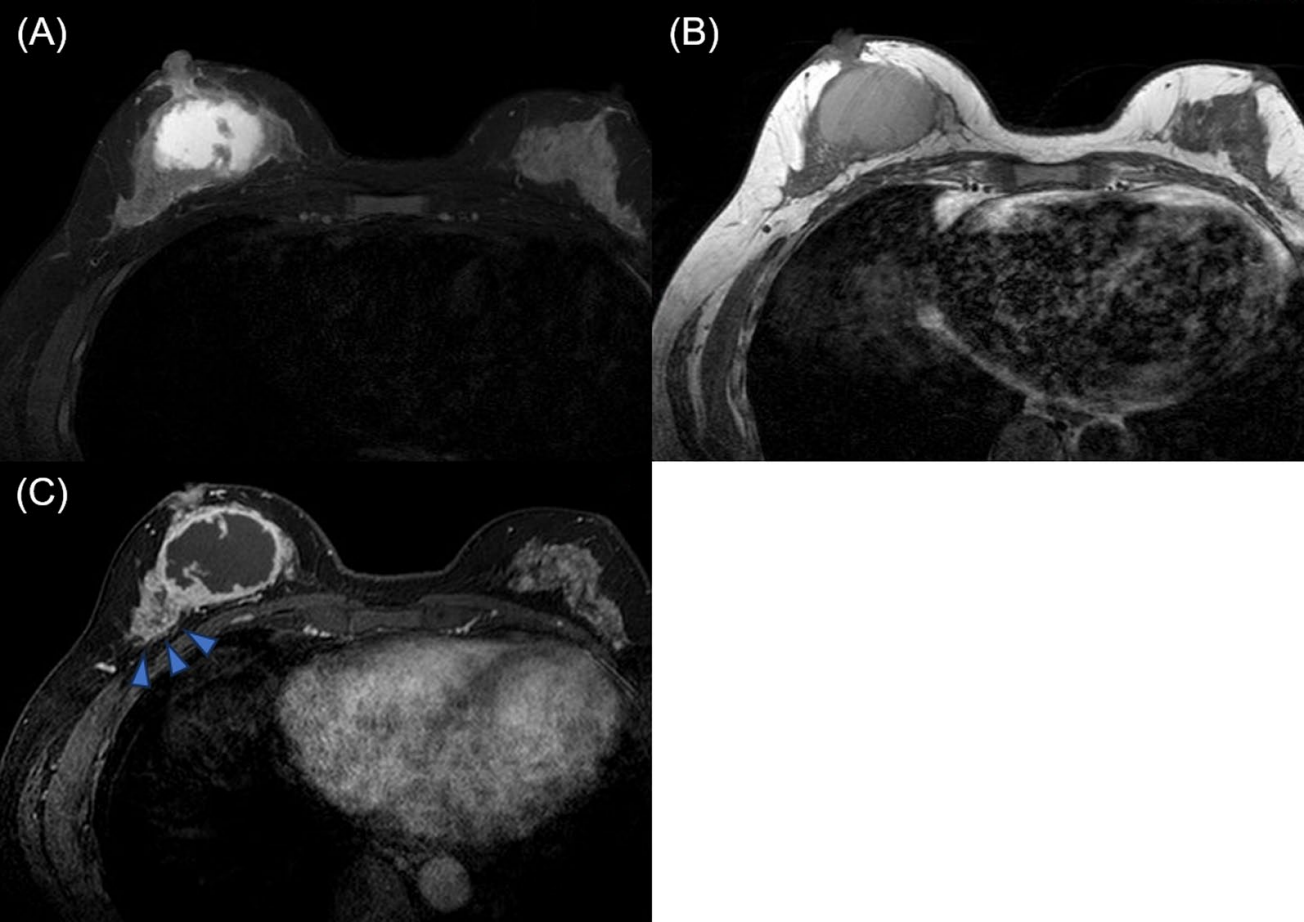
Findings on magnetic resonance imaging. A cystic lesion was observed with high signal on the T2-weighted fat-suppression image (**A**) and high signal on the T1-weighted image (**B**). **C** In the early phase of dynamic contrast enhancement, an irregular spreading of nodule-like staining in the wall was seen, accompanied by broad basal enhancement and bridging (blue arrowheads)

A 43-year-old woman presented to the clinic after feeling a mass in her right breast with associated pain one week earlier. She had no medical history of illness, including trauma. Physical examination revealed a 6-cm elastic, hard mass in the center of the right breast and an enlarged axillary lymph node. There was no obvious redness of the skin. A mammogram showed a 45-mm isodense, partially indistinct, subintra-areolar mass below the right nipple (Fig. 1). Ultrasonography showed a 48 × 45x24-mm cystic mass with an internal broad-based enhancement from just below the right nipple to the lower breast (Fig. 2). Intracystic carcinoma with an invasive component was suspected because the cyst wall was partially obscured and color Doppler showed abundant blood flow. In addition, a hypoechoic area with abundant blood flow was observed directly above the main lesion, suggesting the spread of an intraductal component. One of the level I axillary lymph nodes was enlarged (25 × 23x10 mm), with thick cortical swelling. MRI findings are shown in Fig. 3. The cystic lesion was observed with high signal on the T2-weighted fat-suppression image and high signal on the T1-weighted image, suggesting hemorrhage as the most likely internal fluid component. In the early phase of dynamic contrast enhancement, an irregular spreading of nodule-like staining in the wall was observed, accompanied by broad basal enhancement and bridging, thus an encapsulated papillary carcinoma was suspected. In addition, a non-mass enhancement extended from the cystic wall to the cephalic center, suggesting the spread of intraductal components. In summary, encapsulated papillary carcinoma was most suspected based on the imaging findings.

**Fig. 4:**
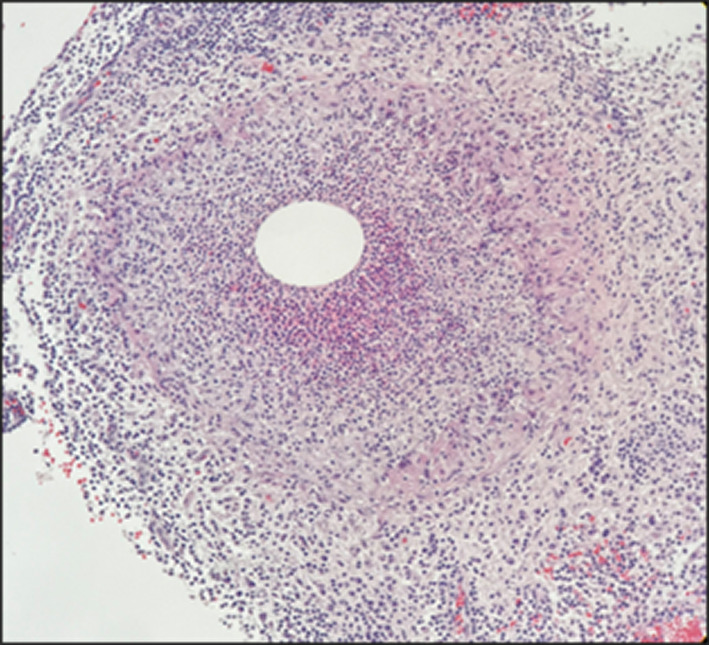
Pathological findings in the vacuum-assisted biopsy specimen. The formation of an epithelial granuloma with a high neutrophilic infiltrate, consistent with granulomatous mastitis (GM), was observed. A circular void at the center is a typical finding in GM

**Fig. 5:**
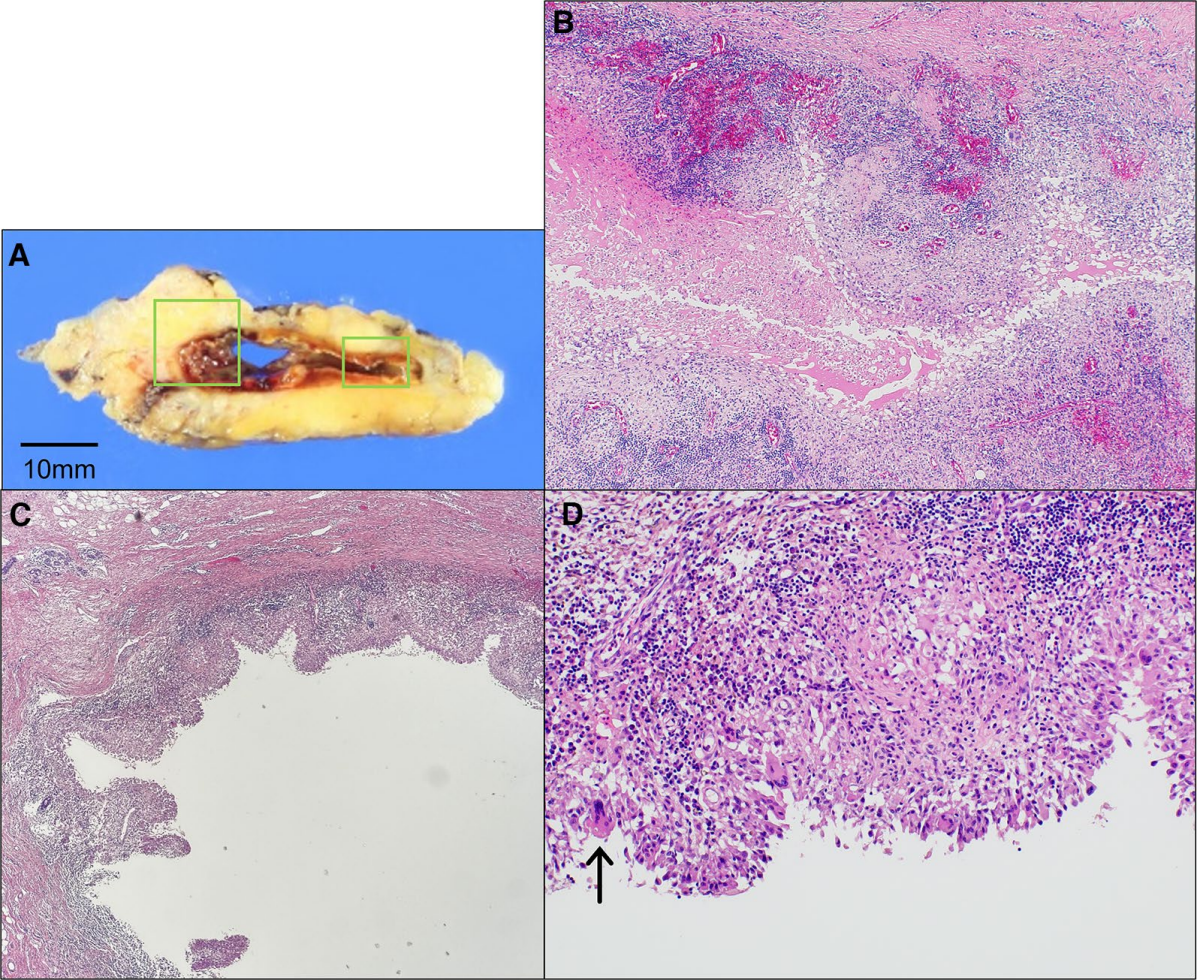
Pathological findings in the surgical specimen. **A** A cystic lesion containing abscess-like contents was observed on the cut surface of the surgical specimen. **B** Enlarged image of the right quadrant in panel A, showing granulomatous inflammation with necrosis and abscess (× 100). **C** Enlarged image of the left square in panel A (× 40). **D** Areas with palisade arrangement of epithelioid cells and a multinucleated giant cell (arrow) are present

A needle biopsy of the breast mass showed granulation tissue with moderate inflammatory cell infiltration. Additional examination with vacuum-assisted biopsy revealed the formation of an epithelioid cell granuloma with a high neutrophilic infiltrate, consistent with a histology of GM (Fig. 4). A ductal component was also observed, but no neoplastic changes were seen. Bacterial cultures were tested but no bacteria were detected. Cytology of the axillary lymphadenopathy was benign. However, since the possibility of malignancy could not be ruled out on imaging, we decided to perform a probe lumpectomy for diagnostic purposes after consultation with the patient. A cystic lesion containing abscess-like contents was observed on the split surface of the surgical specimen (Fig. 5). Histologically, granulomatous inflammation with necrosis and abscess was observed with a large amount of blood. Surrounding microabscesses were also seen, suggesting the diseased areas fused together to form a large mass as the lesion progressed. No neoplastic lesions were found in the specimen, and the final diagnosis was GM. The patient is still free from recurrence of mastitis 22 months after surgery, with only postoperative changes on ultrasonography.

## Discussion

The imaging findings of GM are nonspecific and often overlap with those of malignant lesions. Ultrasonography findings often show an ill-defined hypoechoic mass, and in advanced cases, fluid collection and cavities in association with skin fistulas. Ipsilateral reactive axillary lymph node enlargement may also be present. GM generally has abundant blood flow that can be detected by Doppler imaging within and around the lesion [6]. MRI imaging may show an ill-defined mass or non-mass-enhancement. Often, progressive or plateau enhancement patterns predominate with interspersed areas of rapid enhancement and washout. Advanced cases demonstrate T2 hyperintense, peripherally enhancing masses with central areas of non-enhancement representing abscess formation [12]. In our case, an internal high-signal fluid collection was observed on T1-weighted images, suggesting the possibility of hemorrhage rather than an abscess. In addition, the lesion was a solitary mass, with no surrounding microabscesses, so an intra-cystic tumor rather than GM was the primary concern. As mentioned above, GM generally presents as an irregular shaped mass with ill-defined margins. However, advanced GM cases sometimes demonstrate fluid retention and cavities [5]. Therefore, clinicians should bear in mind that GM can present as a well-defined mass, as in our case.

Although steroid therapy is generally used as a treatment for GM, no treatment method has been established [5, 13]. In clinical practice, long-term steroid therapy is often ineffective, or the disease repeatedly relapses after remission, making it difficult to treat. The efficacy of surgical treatment has long been studied. Postoperative recurrence rates of 14%-25% have been reported when surgical resection is the treatment of choice [10, 14–17]. On the other hand, a meta-analysis comparing the efficacy of steroid therapy to surgical resection reported cure rates of 72%, 91%, and 95% for steroid therapy, surgical resection, and a combination of the two, respectively [18], with relapse rates after remission reported to be 21%, 7%, and 4%, respectively. Although surgical treatment may be overly invasive and should be considered with caution, it should be actively considered as a treatment option in cases of poor response to steroid therapy [10]. In this case the primary goal of surgical resection was to confirm the diagnosis, but as a result, the patient was cured without the use of steroids.

*Corynebacterium kroppenstedtii* has been identified as a cause of GM [19]. It likely triggers granuloma formation through a localized immune response as immune cells attempt to contain the infection [20]. Although often viewed as a contaminant due to its presence in normal skin flora, its deep breast tissue localization within cystic spaces surrounded by granulomatous inflammation indicates a pathogenic role [20]. Hyperprolactinemia, which can cause ductal ectasia and milk stasis, may also promote GM [21]. A combination of these factors might explain why symptoms appear in certain individuals, though further research is needed to fully understand these mechanisms. Detecting *Corynebacterium spp* is challenging due to their slow growth and the need for specific staining, often leading to underdiagnosis. However, it is generally susceptible to various antibacterial drugs; thus, it is essential to make an accurate detection for appropriate treatment.

## Conclusions

We reported a case of GM with imaging findings of a large cystic lesion forming a well-defined bordering mass and a definitive diagnosis was made from a surgical specimen. We believe that our report, particularly such image findings, will be useful for clinicians in daily practice.

## Data Availability

Not applicable.

## Abbreviations

GM: Granulomatous mastitis
MRI: Magnetic resonance imaging
